# Generation of Transplantable Beta Cells for Patient-Specific Cell Therapy

**DOI:** 10.1155/2012/414812

**Published:** 2012-04-26

**Authors:** Xiaojie Wang, Daniel L. Metzger, Mark Meloche, Jianqiang Hao, Ziliang Ao, Garth L. Warnock

**Affiliations:** ^1^Department of Surgery, University of British Columbia, 3100, 910 West 10th Avenue, Vancouver, BC, Canada V5Z 4E3; ^2^Department of Pediatrics, University of British Columbia, 3100, 910 West 10th Avenue, Vancouver, BC, Canada V5Z 4E3

## Abstract

Islet cell transplantation offers a potential cure for type 1 diabetes, but it is challenged by insufficient donor tissue and side effects of current immunosuppressive drugs. Therefore, alternative sources of insulin-producing cells and isletfriendly immunosuppression are required to increase the efficiency and safety of this procedure. Beta cells can be transdifferentiated from precursors or another heterologous (non-beta-cell) source. Recent advances in beta cell regeneration from somatic cells such as fibroblasts could circumvent the usage of immunosuppressive drugs. Therefore, generation of patient-specific beta cells provides the potential of an evolutionary treatment for patients with diabetes.

## 1. Introduction

Type 1 diabetes is one of the most common chronic diseases in children and adolescents caused by autoimmune destruction of insulin-producing beta cells of islets of Langerhans. Thus patients depend on insulin injection all their life. The majority of young patients depend on life-long treatment with insulin injections to control hyperglycemia. However, an exogenous supply of insulin often leads to severe hypoglycemia-related complications. Hence, insulin therapy saves life but is not a cure. On the other hand, beta-cell replacement therapy by transplantation may offer a cure because transplantation of functional beta cells can reestablish glucose-responsive insulin secretion and provide optimal control to prevent hypoglycemia when insulin is secreted [1–9]. Whole-pancreas transplantation can restore endogenous insulin production, but it has rarely been carried out in children with diabetes due to the risk of perioperative morbidity related to the damage by digestive enzymes from the exocrine pancreas during the surgical procedure. In contrast, islet-cell transplantation provides insulin-producing beta cells in a relatively noninvasive manner. It becomes a more feasible option for young recipients. In fact, much progress has been made in islet-cell transplantation following the success of the Edmonton protocol that emphasizes both a sufficient amount of donor islets and steroid-free immunosuppressive regimens [3, 4, 8, 9]. However, the requirement of 2 to 4 donors to reverse diabetes results in a considerable lack of transplantable islets. The destruction of transplanted islets by the cytotoxicity of immunosuppressive drugs further worsens this shortage [7]. In this regard, use of an alternative source of beta cells is a key to bridge the gap between cell supply and demand. Therefore, a major goal of diabetes therapy is to promote the formation of new beta cells. In consideration of elimination of immunosuppressants, autologous cells may offer a safer alternative. Ideally, a patient-specific approach can enhance the success and safety of islet transplantation.

The pancreas is fundamental to the regulation of nutritional homeostasis. The pancreas is composed of exocrine and endocrine compartments. The former consists of acinar and ductal cells that produce and transport digestive enzymes into the duodenum, and the latter of the islets of Langerhans that make hormones for adaptive glucose metabolism. Each islet can secret five hormones (glucagon, insulin, somatostatin, ghrelin, and pancreatic polypeptide), which are produced by alpha, beta, delta, epsilon, and PP cells, respectively [10].

**Figure 1:**
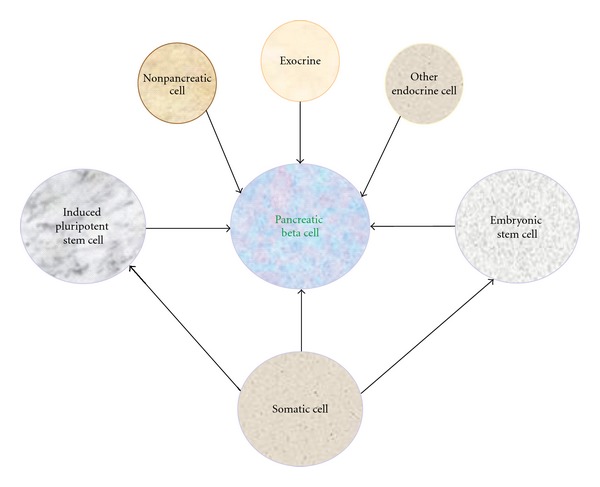
Generation of pancreatic beta cells. New beta cells can be generated by manipulation of different cell sources, such as from other endocrine, exocrine, and nonpancreatic cells, induced pluripotent stem cells, embryonic stem cells, and somatic cells.

There is a great interest in developing novel sources of transplantable beta cells for replacement therapy. Adult beta cells possess a limited capacity to replicate under normal physiologic conditions. However, beta-cell mass expands during times of metabolic changes such as during pregnancy and obesity [11, 12]. Beta cells can also be regenerated after the destruction of existing beta cells, such as by chemical treatment with streptozotocin or the partial removal of pancreas by a surgical procedure [13, 14]. In theory, new beta cells could arise through differentiation of progenitors or other nonbeta cells (Figure 1). Embryonic stem cells have the ability to differentiate into any cell type. For this reason, they are considered as an ideal starting material [15–18]. Some nonpancreatic cells, including hepatic cells, can also differentiate into insulin-positive cells [19, 20]. Non-endocrine pancreatic cells, such as ductal and acinar cells, may retain a degree of plasticity to differentiate into other cell types, including beta cells [21–24]. Beta cells can also be transdifferentiated from other endocrine cells, such as alpha cells [25–27]. 

Recent advances in stem cell biology have established the feasibility of converting one cell type into another [28–31]. This breakthrough directs autologous cell therapy that drives the transdifferentiation of readily available cells, such as fibroblasts, into therapeutically desirable cells, such as blood, neuron, cardiomyocyte, and islet-like cells. Significant applications of such patient-specific therapy include the engineering of new beta cells from patients' own cells, and the elimination of the life-long usage of immunosuppressants, bioincompatibility, and disease transmission coupled with donor cells. Transcription factors for pancreatic stem cell development and the differentiation of beta cell play a critical role in this process.

## 2. Transcription Factors Determine the Development of Beta Cells

**Figure 2:**
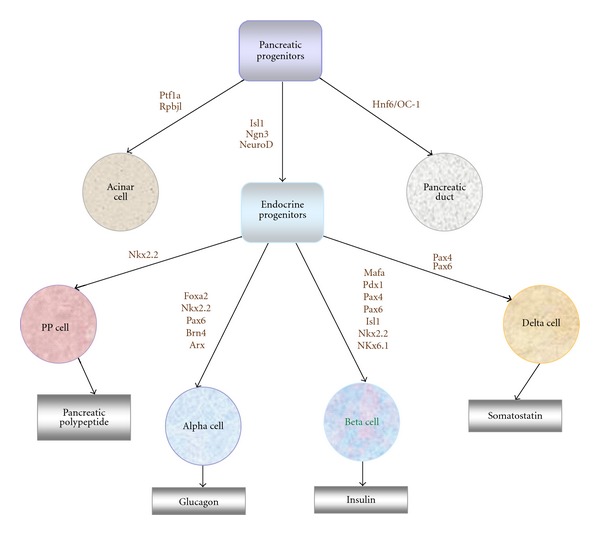
Development of pancreatic beta cells and the associated transcription factors that required for lineage specificity in various steps. Alpha-, beta-, delta-, and pp-cells secrete glucagon, insulin, somatostatin, and pancreatic polypeptide, respectively.

Transcription factors have been recognized as the key mediators of cellular identity. Cell-specific gene expression is controlled at the transcriptional level and in large part by the interface among multiple transcription factors interacting with the promoter and/or enhancer regions of target genes. Many attempts have been made towards generating functional beta cells. The importance of transcription factors in pancreatic development has been well characterized in knockout and transgenic mice using the reverse genetic approach of loss- and gain-of-function of the target genes (Figure 2, [32–35]).

The pancreas develops from dorsal and ventral budding of the foregut boundary beginning around at embryonic day 9 (E9) in mice and at around 3 weeks postfertilization in humans [36]. Both loss- and gain-of-function approaches have confirmed the key role of pancreatic and duodenal homeobox 1 (Pdx1), also known as insulin promoter factor 1, in pancreas development. Genetics lineage tracing has determined the multipotent ability of Pdx1-positive cells, which give rise to all pancreatic cell types [18, 19]. The expression of Pdx1 in abundantly restricted in beta cells in adulthood, although it exhibits a broad and dynamic expression pattern during the developmental stage. The absolute requirement of Pdx1 in normal pancreatic development was well established in Pdx1-deficient mice, which completely lack a pancreas [32, 33]. Furthermore, this the phenotype of pancreatic aplasia was also observed in either heterozygous or homozygous Pdx1 mutations [34]. In humans, mutations in one allele of the Pdx1 gene are associated with adult-onset diabetes (a form of monogenic diabetes), while homozygous mutations in Pdx1 result in pancreatic aplasia [34]. The function of Pdx1 is also demonstrated in studies of gain-of-function. Several studies have shown that nonbeta cells could be transdifferentiated into functional beta cells by overexpression of Pdx1 [25]. Ectopic overexpression of Pdx1 activates pancreatic genes in nonpancreatic cells, and these Pdx1-expressing liver cells can ameliorate streptozotocin-induced diabetes [20]. Therefore, this gene plays an important role in the initial stages of pancreatic development, and it has a great potential as a manipulative target for generating insulin-producing beta cells.

Endocrine versus nonendocrine cell fates appear to be governed by the differential synthesis of the basic helix-loop-helix (bHLH) transcription factors, Ngn3 (neurogenin 3), Hes1 (hairy and enhancer of split 1), and NeuroD1 (neurogenic differentiation 1). Ngn3 is characterized as an endocrine cell-specifying factor, the expression of which is essential for all endocrine-cell development [37, 38]. Lineage analysis of Ngn3-derived cells reveals that all endocrine cell types transiently express Ngn3 during a narrow window in the developing mouse embryo. Ngn3 is detectable at E13.5, peaks at E15.5, and disappears entirely thereafter. No Ngn3-positive cells are found at birth or in adult pancreas, suggesting a role in a particular stage of the pancreas development. In fact, Ngn3-deficient mice proceed along nonendocrine cell fates, confirming its central role as an endocrine progenitor [37, 38]. One key target gene of Ngn3 is NeuroD1, which appears to lie immediately downstream of Ngn3, based on the fact that no NeuroD1 can be found in Ngn3-deficient mice and that expression of Pdx1 is unaffected in NeuroD1 knockout mice. The expression of NeuroD1 can be found in pancreatic endocrine cells and other nonpancreatic tissues, such as the intestine and the brain. The function of NeuroD1 is to activate the insulin gene and drive further differentiation into functional islet cells. In fact, the development of islet cells is arrested at a premature stage in NeuroD1-deficient mice, demonstrating a critical role in early islet formation [39].

The transition from endocrine to individual cell types occurs in two distinct steps. The primary transition features clusters of first-wave glucagon-positive and insulin-negative cells at E9.5. Insulin-positive cells appear during the secondary transition period around E13.5 to E15.5. These cells proliferate and form islet cells soon after birth. The transition from the first to the second stage is not random, but rather each one of the specific endocrine lineages is directed by additional transcription factors. The paired homeobox transcription factors 4 and 6 (Pax4 and Pax6) provide an additional level of specificity for pancreas. Mice deficient for Pax4 display absence of beta and delta cells, suggesting its role in directing the fate of those cells [40]. Pax6 knockout mice fail to develop glucagon-secreting cells, signifying its role in the commitment towards alpha-cell lineage [41].

Arx expression is restricted to islet at mature stage although it is expressed in the pancreas during development. Arx appears to be downstream of Ngn3 based on the fact that it is not expressed in Ngn3-deficient pancreata [42]. Arx-deficient mice die prematurely due to severe hypoglycemia, indicating a possible role in alpha cell development. In fact, a complete loss of alpha cells in the pancreas from Arx knockout mice was observed, confirming that Arx is necessary for alpha cell development [42]. Overexpression of Arx in either Pdx1-positive progenitor cells or insulin-producing cells leads to an increase or conversion in alpha cells, suggesting that Arx is sufficient to turn beta cells towards alpha cell identity [43].

Brain4 (Brn4), a POU domain transcription factor, is only expressed in glucagon positive cells of the adult rat pancreas, suggesting a potential role in alpha cell lineage [44, 45]. Ectopic expression of Brn4 in beta cells leads to the conversion of glucagon-positive cells in both the mantle and the centre of the islet, which led to coexpress insulin and glucagon, suggesting that Brn4 might play a role in early development of glucagon-expressing cells [44]. However, Brn4 is not essential for alpha fate as Brn4-null mutant mice show no significant impact on glucagon or insulin gene expression, biosynthesis, or secretion [45].

The insulin promoter region contains binding sites for both the NK homeobox factors Nkx2.2 and Nkx6.1, indicating that these factors may direct differentiation of insulin-producing beta cells. Nkx6.1 exhibits distinct expression patterns during early and late embryonic stages. It is expressed exclusively in the beta cells by the end of gestation, although it can be stained broadly in the pancreas at E10.5. Mice deficient in Nkx6.1 have no insulin-positive beta cells but are normal for other islet cell types, demonstrating its central role in final differentiation of beta cells [46]. Unlike Nkx6.1, Nkx2.2 can be detected in alpha and PP cells in addition to beta cells. Mice deficient for Nkx2.2 display a complete beta-cell loss and reduced numbers of alpha and PP cells, suggesting its preferential roles in these three cell lineages during pancreatic development [47].

Although certain transcription factors hold specificity, other transcription factors may be required in multiple stages of beta-cell development, and several transcription factors interact with each other to maintain normal pancreatic function. Leucine zipper transcription factors Mafa and Mafb are preferentially expressed within the beta and alpha cells of the adult pancreas, respectively [48, 49]. The transcription of insulin and glucagon is regulated by Mafa and Mafb, respectively. Mice deficient in Mafb exhibit reduced numbers of both insulin- and glucagon-positive cells during pancreatic development, suggesting its essential role in both alpha- and beta-cell differentiation [49]. Mice deficient in Mafa display normal beta-cell development early on, but they develop beta-cell dysfunction and diabetes as adults [48]. This normal phenotype of early beta-cell development in Mafa knockout animals is thought to be due to the compensation from Mafb. Mafb is also found to be expressed in nearly 90% of insulin-producing cells in addition to alpha cells, although this expression ability of Mafb-insulin double positive cells relies on Nkx6.1 expression, in large part due to the lack of insulin-positive cells when Nkx6.1 is knocked down [46, 49]. Forced expression of multiple transcription factors can induce the capacity of differentiation from cell type to another. Experimental data show that combination of the three transcription factors Ngn3, Pdx1, and Mafa directs the reprogramming of differentiated pancreatic exocrine cells from adult mice into islet-like cells [22].

The knowledge on transcription factors is believed to be essential for tailoring the transplantable beta cells to function optimally. First, multiple transcription factors instead of one direct specific cell lineages so that combinatorial interactions obviously are essential for the final pattern of certain cell types. Second, although transcription factors direct the formation of new beta cells, other factors, such as growth factors and transcriptional coactivators are, also needed.

## 3. Endocrine Progenitor Cells

Although the turnover of adult human beta cells is rare, reprogramming may be easier if the starting cell type shares a common developmental history with the desired cell type. The transcription factor Ngn3 directs the formation of the endocrine alpha and beta cells [37, 38]. The development of alpha cells is earlier than that of beta cells. After the endocrine cell types start developing, the glucagons-positive alpha cells become the first to be detectable as early as E9.5. This is followed by the existence of insulin-producing cells, which coexpress glucagon. The fully differentiated beta and alpha cells can be found by E14 [37, 38].

Once fully functional endocrine cells are developed, cell-cell contacts between alpha and beta cells are the greatest among others [25, 26, 37]. For example, the contact rate of alpha and beta cells is much higher than that of alpha-alpha or beta-beta cell contact. This enormous contact between beta and alpha cells suggests a close relationship between these two cell types. In fact, experimental data reveal that alpha cells can serve as a novel source of beta-cell progenitors. Alpha-to-beta cell conversion has been demonstrated independently using different damage models [26, 27]. Detailed studies are needed to clarify the factors mediating the conversion process and the possibility of application to humans.

## 4. Nonendocrine Progenitor Cells

During development, endocrine cells, such as beta cells, and exocrine cells, such as ductal and acinar cells, derive from the same progenitor cells. Adult ductal epithelium has been considered as the most likely source of beta-cell progenitors. This consideration is based on the fact that embryonic insulin-producing cells arise from pancreatic ductal epithelial progenitor cells using morphological staining of beta cells, which are frequently existing in the duct or nearby and also in lineage-tracing studies [24, 50]. It has been found that significant beta-cell neogenesis results from ductal ligation, but this could not be confirmed in other studies [24, 50]. Therefore, the plasticity of ductal cells as progenitors to differentiate into beta cells is still under debate.

Similarly, mouse pancreatic acinar cells can be converted into beta cells using three transcription factors [22]. The newly generating insulin-positive cells were detected by irreversible genetic lineage-tracing techniques and are confirmed to derive from acinar cells [22, 23]. Therefore, both ductal and acinar cells in the exocrine compartment have the potential to be an alternative source for differentiation into beta cells.

## 5. Nonpancreatic Cells

The liver and the pancreas share a common progenitor cell during embryonic development [19, 20]. Therefore, the liver has been examined as an alternative source for generating beta cells [19, 20]. In fact, insulin-positive cells have been produced either in a non-lineage-restricted manner in the presence of high glucose and nicotinamide, or by tissue-specific expression of Pdx1 by adenoviral transfection and with addition of special soluble factors for beta-cell growth [19, 20].

## 6. Inducible Pluripotent Stem Cells (iPSs)

Stem cells possess great plasticity to differentiate into any cell type. However, adult multipotent stem cells are extremely rare and difficult to isolate. Induced pluripotent stem cells (iPSs) are similar to embryonic stem cells and are inducible *ex vivo*. iPSs can be reprogrammed by defined factors from somatic cells [51–54]. Under special conditions, such as the addition of growth factors and multiple transcription factors, iPSs can differentiate into some desired cell types. The significance of this patient-specific conversion would be the avoidance of the risk of immunological rejection.

The desired cell types can be reprogrammed from abundant somatic cells by two major pathways, either directly or indirectly. In the former, beta cells are derived directly from somatic cells, such as fibroblasts [28–31]. They can also be generated through an iPS stage or as the so-called indirect pathway. Novel findings show that islet-like insulin-producing cells can be converted from iPSs, demonstrating the possibility of using patient-specific iPSs as a source of transplantable beta cells for cell-replacement therapies in diabetes. The fact that iPSs can be generated by defined factors using somatic cells makes this strategy possible. Directed reprogramming refers to as direct conversion of beta cells from somatic cells without a multipotent or pluripotent intermediate, mostly directed by a combination of the addition of special growth factors and overexpression of key transcription factors. Fibroblasts can be reprogrammed directly into insulin-producing cells, neurons, cardiomyocytes, and blood-cell progenitors, demonstrating the possibility of converting from one defined cell type to another. Both direct and indirect pathways for regenerating new beta cells have a great impact on patient-specific therapy in the future [28–31]. 

## 7. Conclusion

The success of generating islet-like insulin-producing cell is largely achieved by using our knowledge of the major steps in the differentiation of beta cells during embryonic development of the pancreas. The multiple transcription factors have been applied to coerce available cells to differentiate into desired types in a unique delineation pathway, including across lineages, such as from fibroblasts into iPSs, or from one fully functional lineage to another, such as from fibroblasts into insulin-positive cells. However, major safety issues are still a concern. For example, lentiviruses that are used in the expression of defined factors are unlikely to gain acceptance for transplantation into human patients due to their ability to integrate into the host genome. One of the goals of clinical trials is to balance both safety and efficiency. In terms of safety, some alternate, safer methods, such as the use of nonintegrable viruses, transferring plasmids, soluble proteins of reprogramming mediators, and repeated transfection of mRNA encoding the defined factors, are being tested as an alternative substitute for lentiviruses [52–54]. Although the experiments have been carried out as proof of principle, low efficiency is still an issue for large-scale production in therapeutic applications. Despite the promising progress in the search for alternative sources of insulin-producing beta cells, functionality of new generating cells that replace the same type *in vivo* should be measured considerably. These new transplantable beta cells must mimic extremely closely the function of the normal beta cells if long-term normoglycemia is hoped to be achieved. This is particularly challenging to the production of true insulin-producing cells, due to the enormous complexity of the beta cell. Therefore, it is of great importance to understand in detail the mechanisms that control the origin and fate of beta cells if we hope to reach the ultimate goal of finding a cure for diabetes.

## Abbreviations

Pax: The paired homeobox transcription factors
Pdx1: Pancreatic and duodenal homeobox 1
bHLH: Basic helix-loop-helix
Ngn3: Neurogenin 3
Hes1: Hairy and enhancer of split 1
NeuroD1: Neurogenic differentiation 1
Nkx: The NK homeobox factors.
